# Supplementary material for: Features of the Metabolic Profile of Saliva in Lung Cancer and COPD: The Effect of Smoking Status
Source: Metabolites. 2021 Apr 30;11(5):289. doi: 10.3390/metabo11050289 (PMC8147157; doi:10.3390/metabo11050289)
Supplement: Supplementary file 1 [file metabolites-11-00289-s001.zip › metabolites-1211130-supplementary.pdf]

Table S1. Biochemical indicators of saliva depending on smoking

**Indicators**	**Non-smokers, n=219**	**Smokers, n=169**	**Kruskal-Wallis test (H, p)**
pH	6.48 [6.20; 6.82]	6.50 [6.28; 6.73]	0.0813, 0.7756
Calcium, mmol/L	1.41 [1.03; 1.80]	1.38 [0.98; 1.88]	0.1624, 0.6870
Phosphorus, mmol/L	4.56 [3.40; 5.60]	4.40 [3.27; 5.91]	0.4138, 0.5200
Ca/P-ratio, c.u.	0.33 [0.23; 0.47]	0.30 [0.20; 0.48]	1.646, 0.1996
Sodium, mmol/L	9.0 [5.5; 12.8]	9.6 [6.0; 15.0]	1.213, 0.2708
Potassium, mmol/L	12.5 [9.3; 16.3]	13.2 [9.0; 15.9]	0.7379, 0.3903
Na/K-ratio, c.u.	0.75 [0.47; 1.12]	0.74 [0.48; 1.39]	0.3487, 0.5549
Chlorides, mmol/L	26.5 [21.3; 33.7]	29.5 [22.8; 37.6]	5.775, 0.0163*
Magnesium, mmol/L	0.301 [0.239; 0.377]	0.299 [0.225; 0.369]	0.2250, 0.6353
Protein, g/L	0.67 [0.39; 1.04]	0.59 [0.33; 0.99]	0.9710, 0.3244
Uric acid, μmol/L	90.4 [43.6; 166.7]	79.2 [34.7; 163.5]	0.01516, 0.9020
Sialic acids, mmol/L	0.165 [0.088; 0.287]	0.183 [0.098; 0.281]	0.2644, 0.6071
Pyruvic acid, μmol/L	14.71 [10.54; 22.06]	13.85 [9.31; 21.45]	0.01097, 0.9166
ALT, U/L	4.08 [2.69; 6.31]	3.77 [2.69; 5.38]	0.3394, 0.5602
AST, U/L	5.50 [3.58; 8.08]	5.00 [3.00; 7.17]	2.491, 0.1145
AST/ALT, c.u.	1.32 [1.03; 1.73]	1.22 [0.92; 1.60]	3.603, 0.0577**
LDH, U/L	1158.0 [574.9; 1888.0]	1092.0 [559.5; 1616.0]	0.7354, 0.3911
GGT, U/L	21.6 [17.9; 25.7]	21.7 [18.2; 25.5]	0.01148, 0.9147
Catalase, ncat/mL	2.80 [2.13; 4.33]	2.51 [1.94; 3.50]	4.482, 0.0343*
SOD, c.u.	65.8 [31.6; 130.3]	60.5 [26.3; 100.0]	1.474, 0.2247
α-amylase, U/L	320.1 [173.4; 645.0]	268.2 [124.1; 635.2]	0.4223, 0.5158
AOA, mmol/L	1.69 [1.49; 1.85]	1.85 [1.45; 2.14]	3.563, 0.0591**
Diene conjugates, c.u.	3.98 [3.78; 4.16]	3.98 [3.81; 4.18]	004426, 0.5059
Triene conjugates, c.u.	0.882 [0.779; 0.997]	0.907 [0.792; 0.999]	0.5500, 0.4583
Schiff bases, c.u.	0.556 [0.488; 0.665]	0.551 [0.491; 0.662]	0.00415, 0.9837
MDA, mmol/L	7.35 [5.73; 9.32]	7.01 [5.81; 9.57]	0.06474, 0.7992

Note. * - differences between groups are statistically significant, $p<0.05$; ** - differences between groups are statistically significant, $p<0.10$. AST/ALT – aspartate aminotransferase to alanine aminotransferase ratio, LDH – lactatedehydrogenase, GGT – gamma glutamyltransferase, SOD – superoxide dismutase, AOA – antioxidant activity, MDA – malondialdehyde.

Table S2. Correlation coefficients of salivary biochemical indicators with principal components (Smokers and Non-smokers, Fig. 1b)

Correlation	PC1	PC2	PC3	PC4
Chlorides, mmol/L	0.60	0.42	-0.45	-0.51
AST/ALT-ratio, c.u.	0.42	0.65	0.48	0.42
Catalase, ncat/mL	0.59	-0.46	-0.39	0.53
Antioxidant activity, mmol/L	0.50	-0.50	0.60	-0.37

Table S3. Correlation coefficients of salivary biochemical indicators with principal components (Smokers I, Smokers II and Smokers III, Fig. 1d)

Correlation	PC1	PC2	PC3	PC4	PC5
Phosphorus, mmol/L	**0.80**	0.14	0.14	-0.05	0.42
Potassium, mmol/L	**0.83**	-0.14	-0.14	0.05	0.21
AST/ALT-ratio, c.u.	-0.01	**0.73**	-0.34	0.59	0.01
Superoxide dismutase, c.u.	0.24	-0.02	**0.83**	0.46	-0.21
Ca/P-ratio, c.u.	-0.47	-0.55	-0.10	0.54	0.42
Na/K-ratio, c.u.	-0.58	0.41	0.42	-0.27	0.46

Note. Here and in Tables S4-10, strong correlations are highlighted in red.

Table S4. Correlation coefficients of salivary biochemical indicators with principal components (Smokers I, Smokers II, Smokers III, and Non-smokers, Fig. 1f)

Correlation	PC1	PC2	PC3	PC4	PC5
Phosphorus, mmol/L	**0.79**	-0.01	-0.14	-0.07	0.20
Chlorides, mmol/L	**0.74**	0.05	0.13	0.03	0.43
AST/ALT-ratio, c.u.	0.24	-0.48	0.61	0.50	-0.30
Catalase, ncat/mL	0.19	0.63	-0.26	0.65	-0.28
Superoxide dismutase, c.u.	0.29	0.48	0.47	-0.51	-0.46
Ca/P-ratio, c.u.	-0.40	0.43	0.54	0.17	0.55

Table S5. Correlation coefficients of salivary biochemical indicators with principal components (NO COPD, COPD I and COPD II, Fig.2b)

Correlation	PC1	PC2	PC3	PC4	PC5
pH	-0.47	0.52	0.07	-0.25	-0.05
Calcium, mmol/L	0.44	-0.38	0.50	-0.27	-0.07
Magnesium, mmol/L	0.53	-0.19	0.37	-0.26	0.25
AST/ALT-ratio, c.u.	0.01	-0.07	-0.33	-0.13	0.90
LDH, U/L	**0.71**	0.41	-0.20	0.16	-0.00

Catalase, ncat/mL	0.61	0.57	-0.23	0.09	-0.08
Sialic acids, mmol/L	0.29	-0.57	-0.35	0.24	-0.21
Diene conjugates, c.u.	-0.14	-0.12	0.20	**0.84**	0.20
Schiff bases, c.u.	0.06	0.35	0.68	0.26	0.17

Table S6. Correlation coefficients of salivary biochemical indicators with principal components (NO COPD, COPD I and COPD II + Smokers/Non-smokers, Fig.3b)

Correlation	PC1	PC2	PC3	PC4	PC5
pH	-0.52	0.20	0.01	0.42	0.30
Calcium, mmol/L	0.37	-0.25	-0.56	0.20	-0.56
Chlorides, mmol/L	0.69	-0.12	0.04	0.08	-0.07
Uric acid, µmol/L	0.39	-0.17	-0.29	0.63	0.42
AST/ALT-ratio, c.u.	0.14	-0.02	**0.74**	0.49	-0.34
LDH, U/L	0.52	0.68	-0.08	0.01	0.01
Catalase, ncat/mL	0.31	**0.82**	-0.09	-0.01	0.02
Sialic acids, mmol/L	0.56	-0.36	0.08	-0.22	0.43
GGT	0.66	-0.07	0.33	-0.17	0.08

Table S7. Correlation coefficients of salivary biochemical indicators with principal components (COPD + Smokers I, Smokers II, Smokers III, and Non-smokers, Fig. 4b)

Correlation	PC1	PC2	PC3	PC4	PC5
pH	0.11	-0.18	**-0.76**	-0.27	-0.26
Phosphorus, mmol/L	**0.75**	0.00	-0.13	-0.01	0.12
Uric acid, µmol/L	0.52	0.43	0.29	-0.14	0.04
AST/ALT-ratio, c.u.	-0.04	0.06	-0.18	**0.94**	0.07
Catalase, ncat/mL	0.19	-0.62	0.31	-0.06	0.56
Pyruvic acid, µmol/L	0.48	-0.01	0.44	0.14	-0.59
Diene conjugates, c.u.	-0.11	**0.79**	-0.06	-0.10	0.33
Ca/P-ratio, c.u.	-0.66	-0.01	0.38	-0.11	-0.20

Table S8. Correlation coefficients of salivary biochemical indicators with principal components (COPD Yes/No + Smokers I, Smokers II, and Smokers III, Fig. 4d)

Correlation	PC1	PC2	PC3	PC4	PC5
pH	-0.43	0.53	-0.55	-0.00	0.15
Phosphorus, mmol/L	0.69	-0.08	-0.28	-0.36	-0.12
Catalase, ncat/mL	0.18	0.61	0.31	0.52	-0.43
Pyruvic acid, µmol/L	0.51	0.37	0.06	0.17	**0.73**

Diene conjugates, c.u.	-0.09	-0.57	-0.36	0.69	0.11
GGT, U/L	**0.72**	-0.20	0.23	0.12	-0.02
Ca/P-ratio, c.u.	-0.48	-0.16	0.69	-0.08	0.26

Table S9. Correlation coefficients of salivary biochemical indicators with principal components (COPD Yes/No + Smokers I, Smokers III, and Non-smokers, Fig. 4f)

Correlation	PC1	PC2	PC3	PC4	PC5
pH	-0.30	0.01	0.52	0.33	-0.37
Phosphorus, mmol/L	0.41	-0.71	0.07	0.11	-0.22
Uric acid, µmol/L	**0.73**	-0.04	-0.23	0.07	-0.15
AST/ALT-ratio, c.u.	-0.05	-0.17	-0.27	**0.71**	0.60
Catalase, ncat/mL	0.20	-0.23	0.32	-0.53	0.59
Pyruvic acid, µmol/L	0.68	0.23	0.31	0.19	-0.03
Diene conjugates, c.u.	0.13	0.43	-0.63	-0.14	-0.12
Ca/P-ratio, c.u.	0.22	0.68	0.40	0.14	0.20

Table S10. Correlation coefficients of salivary biochemical indicators with principal components (COPD I, COPD II + Smokers I, Smokers III, and Non-smokers, Fig. 5b)

Correlation	PC1	PC2	PC3	PC4	PC5
pH	-0.65	-0.52	0.06	-0.19	0.16
Calcium, mmol/L	0.61	-0.25	-0.31	-0.16	0.44
Chlorides, mmol/L	0.63	0.07	0.48	-0.09	0.23
AST/ALT-ratio, c.u.	-0.20	0.31	0.39	0.70	0.39
Catalase, ncat/mL	0.33	-0.39	0.34	0.27	-0.67
Diene conjugates, c.u.	-0.07	**0.77**	0.08	-0.41	-0.23
Ca/P-ratio, c.u.	0.13	0.17	-0.76	0.43	-0.15
